# Annotations

**Published:** 1892-04-16

**Authors:** 


### Annotations.

**An Essex Arcadia.**

The Rev. Dr. Jessop has made us acquainted with some of the mental peculiarities of rural East Anglia. Dr. J. C. Thresh, medical officer of health for the Chelmsford and Malden Rural Sanitary Authorities, paints a picture of physical Arcadia in Chelmsford and the neighbourhood which makes one envy the sheep and the cattle that live out of doors the greater part of the year, and that, therefore, need neither villages nor sanitation. Dr. Thresh, in his annual report for 1891 states, among other things, the following: "In Baddow, Springfield, and Ingatestone most of the houses are connected with the sewers. In several other villages the road drains have been converted into sewers, and discharge their contents into ditches or running streams. At Writtle, one of our largest villages, many cottages have been erected recently without drains. The older portion of the village is connected with the old road drains, which were never intended for this purpose, and are totally unsuited for it. The outfall, a ditch, is frequently in a most offensive condition, and the sewage ultimately finds its way into the river and is carried through the town of Chelmsford. In other villages many groups of cottages are drained into ditches, but in too many instances there is no drainage whatever, and the slops are either thrown into the road or into a hole dug in the ground—'a bumby'—at the back of the house. These bumbies receive the house refuse also. They are a constant source of nuisance. The effluvia from the rotting vegetable and animal matters pollute the air, and the filthy fluid percolating through the earth pollutes the ground water, which is frequently the only water available for domestic purposes. There are very few properly constructed and covered ashpits attached to any of the cottages in the district." At one end of the scale in England we have men travelling the same ground over for the ten thousandth time in order to spy out some new, and often trifling fact of science; and at the other end we have members of the same race and the same community herding together in a far worse condition than the bullocks and the sheep, and poisoning themselves and one another, apparently in total ignorance that any such thing as science exists. Happily we have here and there a medical officer of health like Dr. Thresh, who speaks the plain truth in plain words. But of what avail will it be? If we may venture to anticipate next year's report, we prophesy that precisely the same facts will be stated, or that the subject will be altogether avoided. Such is Arcadia in the East of England from a sanitary point of view.

**The Sewage of Isolated Dwellings.**

Country houses and other isolated buildings often fulfil every condition of perfect healthfulness except that of freedom from sewage emanations and poisons. A simple and thoroughly efficient system of drainage for country houses, schools, hospitals, workhouses, and other isolated dwellings is a want which presses upon the attention of thousands of thoughtful and anxious people with painful regularity of recurrence. A few days ago Mr. W. Kaye Parry, member of the Institution of Civil Engineers of Ireland, read a paper at the monthly meeting of the Institute on the subject of the sewage disposal of isolated dwellings. Mr. Kaye Parry showed that he had studied the subject with thoroughness and intelligence. For isolated dwellings the "cesspool system," or some modification of it, is perhaps almost universal. On this point the opinion of Sir John Simon deserves to be seriously pondered. Said Sir John, "The cesspool nuisance has been the slow growth of other less enlightened ages, and it may be taken as an axiom for the purpose of sanitary improvement that every individual cesspool is hurtful to its vicinage." A second principle, which is also of fundamental importance, is quoted from Sir Edwin Chadwick. Sir Edwin laid it down as an absolute rule that "the immediate removal of putrescible excreta before fermentation commences, which is usually in one, two, or three days, is a primary point of sanitation." A third, and equally important, principle maintained by Mr. Kaye Parry is that of "The sewage to the soil." "In all cases," he says, "if land be available, it is far better to apply the sewage to it than to embark upon sewage purification works." We have long contended in this journal that "the sewage to the soil" is the only really rational and profitable method of sewage disposal, and we are glad to find that so intelligent and practical a member of the engineering profession as Mr. Kaye Parry is entirely of our way of thinking, so far at any rate as isolated buildings are concerned.

**Galen's Diagnosis of Love Fever.**

A most interesting lecture on "Galen," by Dr. James Finlayson, of Glasgow, has just been published in the *British Medical Journal.* It bears out our oft-repeated contention that except in the exactitude and completeness of their knowledge, the best physicians of ancient times were not inferior to those of our own day. In intellectual capacity, careful observation, sound experience, and an earnest desire to render their patients the best possible services they were in no sense deficient. Dr. Finlayson's translation of Galen's account of his method of finding out that a particular woman whom he was called in to treat, was not ill, but in love, serves to show to all classes of readers the thoroughness and scientific completeness which were characteristic of the famous physician's work. "I was called," says Galen, "to visit a woman who was troubled with insomnia, and was tossing about on a couch. . . . She was free from fever. . . . I made some enquiries from which I might find out how the insomnia was caused. The woman answered to little purpose. At last, with averted looks, she covered herself up entirely with the bed clothes, and lay with her head turned away on a pillow. I left, and concluded that she was suffering from one of two things—melancholia, or some grief which she was unwilling to avow. . . Next day, on my arrival, I was told by a maid that I could not see her. . . . On the following day I was told the same thing. . . . On the third day the servant told me I might go away, as the woman did not wish to be disturbed. . . . I ascertained that on my departure she had made her toilet and resumed her accustomed ways. . . . When I had made sure that she suffered from no bodily affliction, it happened that at the very time I was visiting her some one coming in from the theatre mentioned that he had seen Pylades dancing. Her look and colour underwent a change; the brachial pulse, which I was holding, became irregular, and suddenly agitated in several ways, the sure index of mental emotion. On the next day I directed one of those who followed me that when I went in on my visit to the woman he would come in shortly after, and mention that Morphus was dancing to day. This was done, but I found no disturbance of the pulse. On the following day I took care that the name of a third dancer should be mentioned, but again there was no alteration of the pulse. On the fourth evening I made a careful experiment: with the pulse in my hand, it was once more mentioned that Pylades had been the dancer. There was now the same agitation as at first, and I concluded that the woman was in love with Pylades, a diagnosis confirmed on subsequent days." This amusing story not only lets us see how very similar were Galen's methods to those of the moderns, but how very much human nature there was in him, in his patient, in her servant, in the assistants who took part in the proceedings, and indeed in everybody concerned.

